# Case control study of CD4 cell count and some haematological parameters among hepatitis and non-hepatitis B patients in Oyo State, South-west, Nigeria

**DOI:** 10.4314/ahs.v23i1.21

**Published:** 2023-03

**Authors:** Adebomi Funmilayo Odusanya, Gabriel Ifeoluwa Makinde, Michael Oladimeji Idowu, Ayodeji Matthew Adebayo

**Affiliations:** 1 University College Hospital Ibadan, Department of Medical Microbiology and Parasitology; 2 University of Ibadan, College of Medicine, Department of Community Medicine

**Keywords:** Hepatitis B virus, CD4 count, hematological parameters, patients

## Abstract

**Aim:**

Hepatitis B virus HBV infection is a major cause of chronic liver disease worldwide. CD4 count and haematological parameters (HPs) could be used to monitor the health status of hepatitis B (HB) individuals. This study aimed at assessing levels of CD4 count and some HPs among sufferers of HB patients and controls.

**Methods:**

Fifty (50) HB patients as cases and 50 age-matched controls were recruited into the study. 5ml of whole blood sample was collected from all eligible participants of which 20µl and 10µl were used for CD4 count and HPs analysis respectively. Pearson correlation analysis was used to assess statistical difference within them using SPSS version 20.

**Results:**

There was significant increase between the normal values of the CD4 count of both cases and controls (p<0.05). Significant correlations were found in some HPs such as HCT with WBC; HB and RBC with PLT; RBC, HCT and PLT with WBC.

**Conclusion:**

There were no significant differences between the values of the CD4 count and hematological parameters among HB subjects in this study. There is need for future studies to detect changes in CD4 count and other HPs in HB patients to increase options of screening for immunological changes during management.

## Introduction

Hepatitis B (HB) is a life-threatening infectious liver disease caused by the hepatitis B virus (HBV). The virus can be transmitted parenterally, perinatally and sexually, and it causes an acute or chronic liver infection which can put people at high risk of death from cirrhosis of the liver and liver cancer^1^.

More than 50% of HB infected persons are without symptoms, meaning that the risk of infecting people unknowingly is very high^2^,^3^. But some people may be suffering from severe symptoms such as jaundice, fatigue, loss of appetite, nausea and/or abdominal pain. In almost all adults, 90% of the infection heals and they become healthy, but for infants and young children, there are 90% and 30 - 50% risk that the infection leads to chronic HB infection. For this group, an approximate 25% increased risk of a later life suffering from liver cirrhosis and/or liver cancer exist, if the infection is not medically managed^3^,^4^,^5^.

In the early stage (within 30 days of infection) of the HBV infection, the viral load is reduced without treatment through the activities of innate immunity^6^. However, in 5-10% of adults, HBV continues to reproduce in the body long after infection. HB can cause chronic infection in which the patients never get rid of the virus and many years later develop cirrhosis of the liver^7^. About one in twenty people with cirrhosis will go on to develop cancer of the liver.

HBV infection is a major cause of liver disease morbidity worldwide, accounting for over 360 million cases of chronic hepatitis and 620,000 deaths per year^8^. According to recent estimates approximately 30% of the world's population; about 2 billion persons have serologic evidence of current or past HB infection^9^. About five percent of the world population are asymptomatic carriers and 350 million people worldwide are chronic carriers of HBV^10^. With the sero-prevalence of hepatitis B virus surface antigen (HBsAg) estimated to range from 10% to 40%^11^, the infection has reached hyper-endemic levels in Nigeria^12^. Nigerian Researchers have found varying HB prevalence rates among different groups. Sadoh et al^13^ reported a prevalence rate of 16.3% among infants, while Oladeinde et al.^14^ documented 5.6% among pregnant women.

A study conducted among pregnant women in Nigeria demonstrated a significant association of HB infection and reduction in haematocrit (HCT), haemoglobin concentration, platelets, total white blood cell, lymphocyte, eosinophil and monocyte counts^15^. Also, Ajugwo et al^16^, and Bozkaya et al^17^ have demonstrated some association between HB infection and abnormal haematocrit, haemoglobin concentration and Erythrocyte Sedimentation Rate; and aplastic anaemia respectively^15^,^16^. Therefore, the varying severity of hepatitis and other liver diseases associated with various stages of HB infection, including the acute and chronic stages, may also produce varying degree of abnormal haematological parameters.

There have been a few documented significant studies and data which demonstrated immunological and hematological responses in HBV and non - HBV infected persons in Nigeria. Clinicians and scientists could argue for inclusion of CD4 cell markers among hematological parameters recommended for follow up monitoring of HB management since it is a major requirement in HIV/AIDS management which is also a viral infection. Hence, this study investigated the CD4 count and some haematological parameters as bio-markers for HB infection in cases and controls. This may provide novel knowledge on screening for clinicians to monitor treatment progress of patients in their quest to deliver HB management care in our community.

## Materials and Methods

### Study Area

The study areas comprise of infectious diseases clinic where patients are cared for, the Haematology and Human Virology Unit (HVU), Department of Medical Microbiology and Parasitology of the Hospital of University College Hospital and Internal Medicine Out-patients clinic where controls were recruited. All these sub-facilities are situated in the University College Hospital; a tertiary and first-class health facility in South-west Nigeria with over 60 years of delivering specialist care that spread across all areas of medicine. The special infectious diseases clinic attends to special infectious diseases such as HIV/AIDS, Lassa fever, Covid 19 etc.

### Sampling technique and study population

Random sampling technique was used to recruit 50 of the patients who had been tested positive for HB over a period of three months. From a pool of 150 patients being managed for HB infection 50 were selected by applying numbers randomly generated from a program in SPSS software. Equal sample size of age matched patients who had been negatively diagnosed for hepatitis were purposively enrolled during same period of recruitment.

Data and samples were collected on the clinic days of consenting patients. The 50 subjects enrolled into respective arm of the study were deduced from prevalence of hepatitis in studies by Mustapha et al^18^. Pro-forma was used to obtain basic information such as age, clinical diagnosis and test results. Additional socio-demographic information such as marital status, educational and employment status were retrieved from patients' medical records. Patients diagnosed with HB infection were eligible for the study. Patients who had HBV with HIV or any other viral infections and those who presented with emergency conditions or those that were too ill were excluded. Patients with no diagnostically confirmed viral infection records were deemed eligible for the study as controls. Cases to controls were age-matched during the recruitment process.

### Data Management

Data were analysed using IBM SPSS package version 20. Descriptive information, means and standard and Pearson correlation analysis were presented in tables.

### Laboratory analysis

5mls of venous blood was aseptically obtained from each patient to establish values of CD4 count and haematology parameters. 20µl of whole blood was dispensed into a corresponding sample tube and placed in PARTEC flow cytometer (Partec GrmbHAM flugplatz 13.D – 02828 Gorlitz. Germany).

To analyse the hematological parameters, 10µl of blood from each participant was taken from the 5ml venous blood and processed in a Sysmex KS-21 N aspirator for all subjects. The generated results were processed, printed for patients and kept for backup and were also saved into designated database.

### Ethical consideration

Ethical clearance which encompasses the Helsinki declaration and its relevant guidelines for research involving humans was obtained from the Joint Ethical committee of the College of Medicine and the University College Hospital Ibadan before the commencement of the study.

## Results

One hundred subjects were enrolled for the study of which fifty were sero-positive for Hepatitis B (HBsAg) virus while the other 50 were the controls.

In both arms of the study, there were more males 75.0% (n=75) than females 25.0% (n=25). The control arm had a higher proportion of male participants 86.0% compared to that of the cases 64.0%. However, more women (37.3%) were recruited into the case arm of the study compared to those in the control (14.0%). The overall mean age of all the subjects was 35.9±10.66 years and 36.0±9.9 and 35.5±11.3 years, respectively for case and controls. There were more married control subjects (86.0%) than cases (76.0%). A higher proportion of control arm participants (64.0%) had secondary school education compared to those in cases arm (52.0%). A larger proportion of cases (76.0%) were employed than the controls (68.0%). Table 1

**Table 1:** Socio-demographic characteristics of subjects

Characteristics	Cases n (%)	Controls n (%)
**Age**		
Mean±SD	35.9±10.66	
**Age categories**		
<36	24 (48.0)	24 (48.0)
≥36	26 (52.0)	26 (52.0)
**Sex**		
Male	32 (64.0)	43 (86.0)
Female	18 (36.0)	7 (14.0)
**Marita status**		
Married	38 (76.0)	43 (86.0)
Single	10 (20.0)	4 (8.0)
Divorced/widow	2 (4.0)	3 (6.0)
**Educational status**		
Primary education	21 (42.0)	15 (30.0)
Secondary education	26 (52.0)	32 (64.0)
Tertiary education	3 (6.0)	3 (6.0)
**Employment status**		
Employed	38 (76.0)	34 (68.0)
Self-employed	10 (20.0)	14 (28.0)
Un-employed	2 (4.0)	2 (4.0)

The mean and standard deviation of hematological parameters of WBC, RBC, HB, HCT, PLT, NEU, LYN, NEU%, MIX% were 5.26±1.85; 4.40±0.65; 12.71±1.65; 37.97±4.59; 199.95±52.14; 2.73±1.57; 44.23±9.27; 51.20±9.99; 4.55±3.56 respectively. Table 2.

**Table 2:** Mean of CD4 cells and other parameters of all subjects

Variables	N	(Mean±SD)	Mean Diff.	p-value
CD4_CELLS	100	894.17±304.54	894.12	0.038
WBC	100	5.26±1.85	5.26	0.008
RBC	100	4.40±0.65	4.40	0.022
HB	100	12.71±1.65	12.71	0.473
PCV	100	37.97±4.59	37.97	0.477
PLT	100	199.95±52.14	199.95	0.001
NEU	100	2.73±1.57	2.73	0.112
LYN	100	44.23±9.27	44.23	0.633
NEU_PERCENT	100	51.20±9.99	51.20	0.188
MIX%	100	4.55±3.56	4.55	0.006

The CD4 cells of both cases and controls were within normal range. However, the mean CD4 count of HB patients were significantly higher than that of the controls (p=0.038). Also, the mean of other hematological parameters irrespective of the enrolment status were within normal range. However, the mean of parameters such as WBC, RBC, PLT and MIX% were significantly higher in the cases than in controls (p<0.05). Table 3.

**Table 3:** Mean of CD4 cells and other parameters of Hepatitis B patients and controls

Variables	Cases (Mean±SD)	Mean Diff.	p-value	95%CI	Controls (Mean±SD)	Mean Diff.	p-value	95%CI
CD4 CELLS	956.27±308.9	125.46	0.038	7.20–243.71	830±289.50	125.46	0.038	7.28–243.63
WBC	5.735±2.23	0.96	0.008	0.25–1.67	4.77±1.20	0.96	0.008	0.25–1.67
RBC	4.5443±0.74	0.30	0.022	0.04–0.55	4.25±0.51	0.30	0.022	0.04–0.55
HB	12.825±1.75	0.24	0.473	-0.42–0.89	12.59±1.56	0.24	0.473	-0.42–0.89
PCV	38.29±4.91	0.65	0.477	-1.16–2.47	37.64±4.26	0.65	0.476	-1.16–2.47
PLT	216.08±54.88	32.58	0.001	12.93–52.23	183.50±43.92	32.58	0.001	12.96–52.19
NEU	2.9842±2.04	0.50	0.112	-0.12–1.12	2.48±0.83	0.50	0.113	-0.12–1.12
LYN	44.67±10.22	0.89	0.633	-2.79–4.56	43.78±8.26	0.89	0.632	-2.78–4.56
NEU%	49.88±10.99	-2.64	0.188	-6.59–1.31	52.52±8.81	-2.64	0.188	-6.59–1.31
MIX%	5.55±4.15	1.96	0.006	0.57–3.35	3.59±2.57	1.961	0.007	0.55–3.37

Normal values of CD4 and other hematological parameters

CD4= 500–1500s cells per milliliter

WBC= 4000–11,000 cells per cubic milliliter

RBC= 4.2–6.1 million per micro-liter

HB=12–18g/dl

PCV=37–53%

Platelets=150,000–450,000 per micro-liter

Total neutrophil count=2500–7500 micro-liter

Neutrophil=40–60%

Lymphocyte=20–40%

Monocytes=2–8%

The white blood cell counts in both groups and the haemoglobin count in the controls statistically had positive correlation with CD4 count of the patients. p<0.05. The neutrophil count had significant negative correlation with the WBC parameter of patients in both groups. However, HCT and NEU% had positive correlation with the cases arm only. p<0.05. HB and HCT counts of both cases and controls had positively significant correlations with RBC count of the patients. p< 0.05.

The platelet counts had significant positive and negative correlation with HB and RBC counts of HBV patients, whereas, the CD4, WBC and lymphocyte counts showed positive correlation with platelet counts of those without the ailment. p<0.05. There were consecutive significant negative and positive correlation between LYN against NEU counts and Neutrophil versus NEU% counts in both arms of the study. p<0.05. Negative significant correlation existed between NEU% and RBC counts among the HBV patients. p<0.05. There was a significantly negative correlation between NEU% counts and MIX% counts in the control arm of the study. p<0.05. Table 4.

**Table 4:** Correlation of CD4 counts with other haematological parameters and correlations between the parameters

Variable	Statistics	Cases	Controls
CD4↔**WBC**	R	0.344	0.543
	p-value	0.013	0.000
NEU↔**WBC**	R	0.944	0.056
	p-value	0.000	0.000
HCT↔**WBC**	R	-0.283	0.024
	p-value	0.044	0.871
HB↔**RBC**	R	0.597	0.766
	p-value	0.000	0.000
HCT↔**RBC**	R	0.848	0.846
	p-value	0.000	0.000
NEU↔**HCT**	R	-0.286	-0.045
	p-value	0.044	0.756
HB↔**PLT**	R	0.339	0.173
	p-value	0.015	0.229
RBC↔**PLT**	R	-0.339	0.278
	p-value	0.015	0.051
WBC↔**PLT**	R	0.153	0.393
	p-value	0.284	0.005
LYN↔**NEU**	R	-0.694	-0.426
	p-value	0.000	0.002
NEU%↔**NEU**	R	0.763	0.465
	p-value	0.000	0.001
RBC↔**NEU%**	R	-0.310	-0.221
	p-value	0.028	0.123
NEU%↔**MIX%**	R	-0.194	-0.315
	p-value	0.192	0.027

## Discussion

Hepatitis B virus is a major cause of chronic liver disease worldwide and is one of the diseases of mankind that has been considered as a serious global health problem. The World Health Organization (WHO)^19^ advocates HB testing especially in areas of high HB prevalence but additional evaluation of haematological parameters and testing for HbsAg markers such as HBeAg and HBV DNA and tests to assess stage of liver disease (e.g., liver enzymes, liver biopsy, etc) might not have been effectively optimized in management or not widely available in many resources limited countries^20^.

Findings from this study revealed that CD4 cells mean significantly increased among HB patients than controls. It was expected that the reverse would be the case. Longitudinal studies aimed at investigating the possibility of CD4 profile reduction based on different levels of mono-infected HBV patients may unravel a connection between the two.

This current study found significant statistical difference in some hematological parameters between cases and controls and within the sex of the participants. Significant parameters identified among cases and controls include red blood cells count and platelet count while the sex specific significant parameters were PCV and HB. However, these findings were mostly between normal and above normal values for all the patients. The aetiology of platelet count changes in patients with liver fibrosis is complex, but one of the possible causes suggested may include HBV virus which could affect bone marrow megakaryocytes and cause thrombocytopenia. Also, liver fibrosis affects the production of thrombopoietin, but studies have found no significant relationship between platelet count and thrombopoietin concentrations^21^,^22^.

Previous studies have established that there is haematological abnormality at the acute stage of hepatic viral infection^21^, hence a full blood count to exclude bacterial sepsis as a cause of liver disease and other non-specific symptoms which may mimic other more common infections seen in sub-Saharan Africa are often recommended. Alteration in the blood count may exclude to some reasonable extent the presence of these other infections^23^. To make preliminary inference from the relationship between abnormal haematological outcomes, HbsAg patients might be required to continue periodic diagnosis for due observation into the patterns of subsequent test outcomes. This will not only underscore the need for a full blood count in all patients with acute viral hepatitis, it will also help to identify those patients that will need to be followed up or require additional treatment. Viral hepatitis is a pan tropic disease with haematological manifestations^24^. Alteration in haematological profile may predict those likely to have haematological complications even after recovery from the acute viral hepatitis^24^.

With reference to the haematological findings of this study, follow-up repeat of haematological investigations in a longitudinal study would be helpful in ascertaining and affirming specific haematological parameters that are critical to the management of HBV infection.

This study found intrinsically positive significant correlation between some haematological parameters of HBV positive patients. These include correlation of; HCT and NEU with WBC; HB and HCT, with RBC; HB and RBC with PLT and HCT and LYN, with NEU. Correlation studies in this direction are scarce however, literature have established some influence of HBV on some haematological parameters. Leucopaenia rather than leucocytosis is a more frequent finding in acute viral hepatitis, though leucocytosis is often associated with fulminant viral hepatitis^25^. The findings that leucocyte is another non-hepatic site for HBV replication which causes direct invasion of the marrow thus interfering with leucopoiesis supports the more frequent finding of leucopaenia rather than leucocytosis

Significant higher mean levels of serum bilirubin and higher proportions of prolonged prothrombin time have been found in HB patients with anemia, thrombocytopenia or leucocytosis, suggesting that these hematological abnormalities were closely related to the severity of hepatocellular damage^27^. With respect to platelet count, mild to moderate thrombocytopaenia is not unusual in patients with acute viral hepatitis. HCV antibody positive individuals are 2.6 times more likely to have low platelet count than those who are HCV antibody negative^27^.

## Conclusion

The CD4 level reduction and haematological change in patients with viral hepatitis has not been fully proven to be specific as this study still found normal values of CD4 cells and hematological parameters in the subjects of this study.

## Limitations

Sample selection bias was likely to have occurred during recruitment of patients through use of simple random sampling. This was because admission of patients into the clinic was ongoing thus sampling was only done for limited number of patients on records during the period of recruitment. In other words, there could have been more cases to draw a representative sample from. This could lead to skewness of full population of interest making additional sampling techniques a requirement in this scenario.

Since the study did not endeavour to select representative controls then the measure of association of this study was likely to be biased i.e., over-reported or under-reported.

## Recommendations

Patients would still need to be followed up for early detection of serious complication by continued evaluation of hematological parameters even after recovery. There is need for further expanded research founded on relevant eligibility criteria to provide concrete and empirical evidence linking HbsAg to decline in CD4 cells alongside other hematological parameters.
